# Apathy Is Associated With Reduced Precision of Prior Beliefs About Action Outcomes

**DOI:** 10.1037/xge0000739

**Published:** 2020-02-10

**Authors:** Frank H. Hezemans, Noham Wolpe, James B. Rowe

**Affiliations:** 1 Medical Research Council (MRC) Cognition and Brain Sciences Unit and Department of Clinical Neurosciences, University of Cambridge

**Keywords:** apathy, motivation, goal-directed action, Bayesian, sensorimotor prediction

## Abstract

Apathy is a debilitating syndrome that is associated with reduced goal-directed behavior. Although apathy is common and detrimental to prognosis in many neuropsychiatric diseases, its underlying mechanisms remain controversial. We propose a new model of apathy, in the context of Bayesian theories of brain function, whereby actions require predictions of their outcomes to be held with sufficient precision for “explaining away” differences in sensory inputs. In the active inference model, apathy results from reduced precision of prior beliefs about action outcomes. We tested this hypothesis using a visuomotor task in healthy adults (*N* = 47), with experimental manipulation of physical effort and financial reward. Bayesian modeling of performance and participants’ perception of their performance was used to infer the precision of their priors. We confirmed that the perception of performance was biased toward the target, which was accounted for by relatively precise prior beliefs about action outcomes. These priors were consistently more precise than the corresponding performance distribution, and were scaled to effort and reward. Crucially, prior precision was negatively associated with trait apathy, suggesting that apathetic individuals had less precise prior beliefs about action outcomes. The results support a Bayesian account of apathy that could inform future studies of clinical populations.

Apathy is common, debilitating, and detrimental to the prognosis in many neurological and psychiatric diseases (Lanctôt et al., 2017; Lansdall et al., 2019; Starkstein, Jorge, Mizrahi, & Robinson, 2006), but it also occurs to varying degrees in the healthy population (Ang, Lockwood, Apps, Muhammed, & Husain, 2017). Apathy is a complex construct, often decomposed into emotional, cognitive, and behavioral domains (R. Levy & Dubois, 2006; Robert et al., 2009). However, its underlying mechanisms are controversial and several accounts have been put forward for the reduction in “goal-directedness” of behavior that characterizes apathy.

Behavioral economics and reinforcement learning models cast apathy primarily as a pathology of value-based decisions (Husain & Roiser, 2018). On this basis, apathetic individuals behave in ways that fail to maximize their utility, given information about the likely costs and benefits of different actions. Specifically, they exert less effort for reward (Chong, Bonnelle, & Husain, 2016), which has been attributed to deficits in dopamine-dependent reward sensitivity (Adam et al., 2013; Le Bouc et al., 2016; Muhammed et al., 2016). However, defining apathy as a lack of dopamine-dependent motivation has limitations. Current paradigms constrain action to be the consequence of a stimulus (such as a reward cue) and subsequently evaluate the action against an external reward function. This does not directly address the subject’s desire to actively fulfil their internal goals and beliefs or expectations (Gottlieb & Oudeyer, 2018). Goal-directed behavior can alternatively be regarded as anticipatory rather than reflexive, such that actions are driven by their intended consequences (Hommel, Müsseler, Aschersleben, & Prinz, 2001; Prinz, 1997).

Here, we propose that apathy is directly related to the dependence of motivated behavior on the precision of the representations of internal goals and beliefs about action outcomes. We build on the concept that brain function is a form of hierarchical Bayesian inference (Clark, 2013; Hohwy, 2013). On this basis, the brain maintains a generative model that optimizes predictions of sensory inputs and minimizes prediction error or “surprise” (Friston, 2010; Friston, Daunizeau, Kilner, & Kiebel, 2010). Prediction error can be minimized in two ways: passively, by changing predictions to better fit the sensory inputs (perceptual inference), or actively, by performing actions to change the sensory input itself (active inference; Adams, Shipp, & Friston, 2013; Friston et al., 2010). We propose that apathetic behavior is a disorder of active inference, within an enactive Bayesian framework.

The key question with regard to apathy is how the balance between perception and action is regulated. Under active inference theory, the precision (inverse uncertainty) of predictions and sensory input determine their relative contribution to behavior. When predictions are held with high precision, they will be maintained even in the face of conflicting sensory input, and induce action so that the predicted and current state of the world are no longer in conflict. This means that action requires sensory attenuation: the transient down-weighting of sensory prediction errors so that expectations and goals can be fulfilled through action (Brown, Adams, Parees, Edwards, & Friston, 2013; Wolpe et al., 2016; Wolpe, Nombela, Ingram, & Wolpert, 2018). Thus, a driving force of action is the regulation of the precision of predictions. A corollary is that low prior precision leads to a more passive behavioral state, where prediction errors are resolved by changing prior beliefs about the environment instead of by action (Friston et al., 2010, 2014).

The precision of prior beliefs can be inferred through computational modeling of behavioral data (e.g., Wolpe, Wolpert, & Rowe, 2014). This can be used to test the mechanism of individual differences in apathy, in healthy adults and clinical populations, and in relation to clinical outcomes and neural data (Adams, Huys, & Roiser, 2016). In the context of visuomotor tasks, we previously found that the precision of priors was associated with trait optimism, such that more optimistic individuals tended to have more precise priors, leading to a perceptual distortion toward better performance (Wolpe et al., 2014). Evidence from Parkinson’s disease suggests that the precision of priors is related to dopamine (Wolpe, Nombela, & Rowe, 2015).

To bring these separate lines of evidence into a common analytical framework, we hypothesized that individuals with greater apathy have less precise prior beliefs about their action outcomes. We tested this hypothesis using a visuomotor task that independently manipulated effort and reward, and from which the precision of action priors could be estimated psychophysically. We predicted that participants’ estimates of performance would be biased, in line with the integration of sensory evidence with prior beliefs about action outcomes. Using Bayesian modeling of the participants’ performance and their reported perception of performance, we estimated the precision of participants’ priors and its fluctuation across levels of effort and reward. We tested whether individual differences in apathy are related to variation in the precision of priors, and how the precision of priors depends on effort and reward.

## Method

### Participants

We aimed to be sufficiently powered to detect moderate associations between task metrics and apathy as follows: to detect a true correlation of ρ = 0.4 with α = .05 (two-tailed) and power of 80%, the required sample size is 46. We recruited 53 healthy adult participants to account for at least 10% data exclusions from aberrant performance profiles or technical issues. The participants had no history of a neurological or psychiatric disorder and had normal or corrected-to-normal vision. The study was approved by the Cambridge Psychology Research Ethics Committee, and all participants provided written informed consent. Participants received a standard compensation of £6 per hour and a bonus of up to £5 based on performance. Participants completed the Apathy Motivation Index (AMI; Ang et al., 2017), a questionnaire measure of apathy that is designed for the healthy adult population.

We excluded five participants whose average task performance was ≥3 times the median absolute deviation from the group median performance, and one participant who could not perform the force calibration appropriately. The reported analyses are therefore based on 47 participants (24 females, age range of 18–35 years, *M* = 24.75, *SD* = 4.79; further demographics are given in Supplementary Table 1 in the online supplemental materials).

### Task and Procedure

**Figure 1 fig1:**
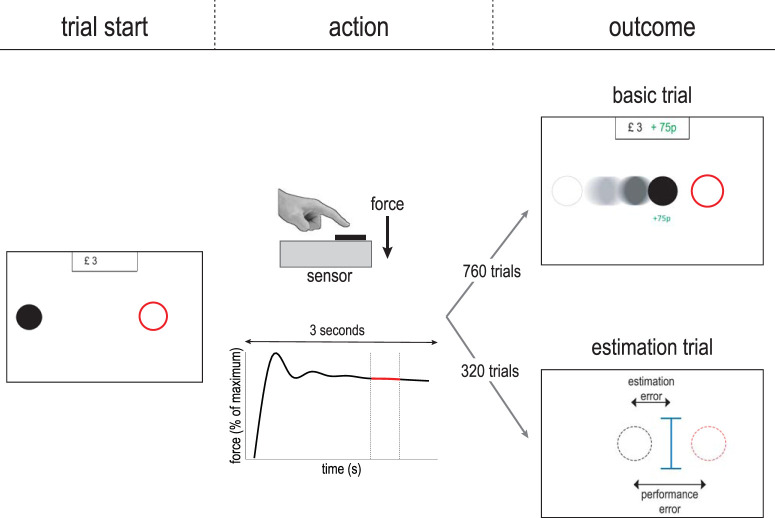
Overview of the visuomotor task. Participants performed a sustained finger press to trigger a ballistic ball trajectory, aiming it at a target. The target was displayed either close to or far from the ball’s start position, corresponding to 35% (low effort) or 65% (high effort) of the participant’s maximum force. Further, participants were given either a performance-dependent monetary reward or no reward. For a minority of trials, the ball’s movement trajectory was not displayed, and participants estimated the ball’s final position with a cursor.

The visuomotor task (see Figure 1) was designed to infer the precision of prior beliefs and its influence on the perception of action outcomes, under different levels of effort and reward. Participants pressed a force sensor to control the subsequent ballistic trajectory of a “ball” cursor on the screen (32-pixel radius). The aim of each trial was to “land” the cursor on the target (38-pixel radius). The target was either displayed close (512 pixels from left margin) or far (896 pixels from left margin) from the ball’s start position (128 pixels from left margin), such that the distance to travel corresponded to 35% (low-effort condition) or 65% (high-effort condition) of each participant’s maximum force. Performance was either rewarded (in “points”, to be converted to cash reward after the study) or not rewarded (reward or no-reward condition).[Fig-anchor fig1]

For each trial, participants performed a sustained finger press for 3 s, after which the black ball turned green to indicate that the finger could be released. Within the 3-s recording, we took the mean force from 2 to 2.5 s as the response (Wolpe et al., 2016). The force response determined the initial velocity of the ball. The deceleration of the ball was constant, and therefore the initial velocity (i.e., force response) uniquely determined the ball’s final position. The difference between the force response and the force needed to land the ball perfectly on target constitutes the force error, expressed as a percentage of the participant’s maximum force.

#### Trial type

The task consisted of two types of trials: basic and estimation. For basic trials, participants viewed an animation of the ball’s trajectory from the start position to the final position in the direction of the target—that is, the outcome of their action. The difference between the ball’s final position and the target constitutes the performance error, expressed in pixels. For estimation trials, the ball’s trajectory was hidden and participants used a mouse cursor to provide their estimate of where the ball would have finished. The difference between the estimated final ball position and the true final ball position constitutes the estimation error, expressed in pixels. Note that the target was not displayed during the estimation procedure, and participants did not receive any feedback regarding the true final ball position. Furthermore, participants were not precued about what type of trial they were engaging in. For estimation trials the ball’s animation started as usual, but after traveling 10% of the screen width, the screen turned blank and the cursor was drawn to the screen.

The experiment started with two practice blocks of 50 basic trials each, with the target in the center of the screen (704 pixels from the left margin). In the second practice block, participants were asked to estimate their performance after viewing the full trajectory of the ball, to introduce the estimation procedure. The test phase consisted of 40 blocks of 27 trials each. We used a 2 × 2 full-factorial design (low and high effort; no reward and reward). In the reward condition, the maximum score of £1 was given when the ball landed perfectly on the target, and this score decreased linearly as performance error increased. To avoid confounding the effort and reward manipulations, the minimal performance required for a reward was more stringent in the low-effort condition than in the high-effort condition (15% of the screen width from the close target vs. 30% of the screen width from the far target).

There were 10 blocks of trials for each combination of effort and reward, and the blocks were ordered pseudorandomly for each participant at the start of the experiment. Each block consisted of 19 basic trials and eight estimation trials. The trial order within each block was determined pseudorandomly, with the constraints that the first three trials were always basic trials, and that there could never be two consecutive estimation trials. Overall, excluding practice, participants completed 1,080 trials, of which 320 were estimation trials. To reduce fatigue effects, we gave participants the opportunity to take a short break after completing a block.

#### Maximum force calibration

At the start of the task, we established each participant’s maximum force to normalize the effort levels between participants. This procedure consisted of three trials of 10 s each. Participants pressed with the maximum level of force they could sustain for the duration of the trial, using the index finger of their dominant hand. At the end of each trial, a sliding window function was used to select the 5-s window with the lowest force variance, and the mean force within that window was taken as the maximum force for that trial. The highest value across trials was taken as the participant’s true maximum force.

The maximum force was used to convert the force response to the ball’s initial velocity. The applied force was divided by 25% of the maximum force and then multiplied by 30% of the screen width per second. That is, pressing at 25% of one’s maximum force caused the ball to initially move at 30% of the screen width per second. To make the task less difficult under higher levels of force, we also scaled the relationship between force and initial velocity by multiplying the applied force by 0.5.

### Data Analysis

#### Task performance

We preprocessed the data as follows: (a) we removed the first trial from each block to exclude any effects of switching between experimental conditions, which reduced the total number of trials from 1,080 to 1,040 (260 per condition); (b) for each participant and each condition, we removed trials with a force error that was more than three times the median absolute deviation away from that condition’s median. On average, we removed three trials per condition for each participant.

We first examined the effects of effort and reward on behavioral performance. Accuracy (median force error) and variability (interquartile range of force error) served as dependent variables in repeated-measures analysis of variance, with effort and reward as within-subjects factors. We report generalized eta-squared (η̂_G_^2^) as the estimate of effect size, and we performed post hoc Tukey’s tests to compare levels of effort and reward. We also performed Bayes factor (BF) analyses with the default JZS prior to quantify the relative evidence in favor of a model, given the data (Rouder, Morey, Speckman, & Province, 2012).

#### Precision of prior beliefs

To infer the precision of prior beliefs in the perception of action outcomes, we examined participants’ estimates of their own performance as follows. For each estimation trial, we assumed that the prior and sensory evidence are Gaussian, such that the optimal estimate of the ball’s final position can be derived from Bayes’s rule (Wolpe et al., 2014):

$$x_{estimate}=w\cdot \mu_{prior}+(1-w)\cdot \mu_{evidence},$$ 1

where the weighting *w* is given by

$$w=\frac{\sigma_{evidence}^{2}}{\sigma_{evidence}^{2}+\sigma_{prior}^{2}}.$$ 2

For a given estimation trial, we consider the ball’s true final position as the mean of the sensory evidence distribution μ_evidence_, and the target position as the mean of the prior distribution μ_prior_. If a participant has no clear prior expectation regarding their performance (i.e., a “flat” prior with very large variance $\sigma_{prior}^{2}$), the estimate of the ball’s final position would be similar to the ball’s true final position, affected only by sensory noise with variance $\sigma_{evidence}^{2}$. Conversely, if a participant has an exaggerated expectation of success (i.e., a prior with very small variance $\sigma_{prior}^{2}$), this prior would “overwhelm” the sensory evidence, leading to estimates of performance that are biased toward the target relative to the true final ball position.

#### Relative weighting of priors

The first equation can be rewritten as

$$\underset{estimation\;error}{\underset{︸}{x_{estimate}-\mu_{evidence}}}=-w\cdot \underset{performance\;error}{\underset{︸}{\left(\mu_{evidence}-\mu_{prior}\right)}},$$ 3

where the slope of a linear regression of estimation error by performance error characterizes the weighting term *w* (Vilares & Kording, 2017; Wolpe et al., 2014). A slope of −1 corresponds to full reliance on priors relative to sensory evidence, whereas a slope of 0 corresponds to a disregard of priors relative to sensory evidence.

We used linear mixed models to fit the linear relationship of estimation error by performance error. As a baseline model, we allowed the intercept and slope to vary by participants. Given that we expected performance error to depend on effort and reward, we also fit a set of models with an additional random effect term to allow for adjustments by effort and reward within each participant. Specifically, within each participant we allowed either the intercept, the slope, or both to vary by either effort, reward, or both, resulting in nine additional linear mixed models. For each model we retrieved the conditional Akaike information criterion (AIC) as an approximation to the log model evidence. We selected the model with the lowest conditional AIC value as the most parsimonious model.

#### Modeling of prior precision

To estimate the prior precision for each participant, we fit the data with a set of hierarchical Bayesian models. The first model assumed that the prior distribution was centered on the target with unknown variance $\sigma_{prior}^{2}$, and the sensory evidence distribution was centered on the true final ball position with unknown variance $\sigma_{evidence}^{2}$. The observed estimates of the ball’s final position were then modeled as a precision-weighted combination of the prior distribution and the sensory evidence distribution (see Equation 1). However, performance of the task may have been affected by computational imperfections (Stengård & van den Berg, 2019), such as perceptual shifts as a result of the ball’s rightward motion or a general bias toward the center of visual space. The second model therefore featured an additional free parameter, s, to account for directional shifts in the mean of sensory evidence:

$$\mu_{evidence}=x_{true}+\text{s}.$$ 4

Although we consider the target as the mean of the prior distribution, participants could instead use “observational” priors that reflect their actual performance distribution. We therefore additionally fit the data with a model that was similar to the first model, except the prior distribution was determined by the mean and standard deviation of each participant’s true performance on basic trials (i.e., when the true final ball position was shown).

We estimated the free parameters hierarchically: (a) parameters for individual participants were considered samples from group-level Gaussian distributions and (b) within each participant, parameters were permitted to vary between experimental conditions. Further details about the model specification are provided in Figure 5A and Supplementary Figure 1 in the online supplemental materials.

We used Markov chain Monte Carlo sampling to approximate the posterior distributions of parameters simultaneously at the level of the group, participant, and conditions. For each model, we used eight independent chains with 2,000 samples, discarding the first 1,000 samples as the “burn-in” period. We assessed model convergence by the chains’ time series plots, and confirmed that the potential scale reduction statistic *R̂* was less than 1.01 for all parameters. To identify the best model, we computed the Widely Applicable Information Criterion (WAIC) to estimate each model’s pointwise predictive accuracy, penalized for the effective number of parameters (Vehtari, Gelman, & Gabry, 2017). Our primary interest was in the participant-level estimates of prior precision, as well as the change in prior precision across levels of effort and reward.

#### Prior precision and trait apathy

We tested the relationship between model estimates of prior precision and individual differences in trait apathy. We measured trait apathy with the AMI, a questionnaire measure of apathy that is suitable for the healthy population and has strong psychometric properties (Ang et al., 2017). The AMI provides a mean total score as well as mean scores for three different domains of apathy: behavioral activation, emotional sensitivity, and social motivation. Each subscale consists of six Likert-type scale items scored from 0 to 4, where higher scores indicate greater apathy.

As action priors correlate with performance variability and to rule out the effect of performance, we computed the partial correlation between trait apathy and prior precision using Pearson’s correlation, adjusted for individual differences in performance variability. Specifically, we used each participant’s standard deviation of performance error for all basic trials as an index of performance variability. We performed the partial correlation analysis separately for each outcome of the AMI. We also report the BF for partial correlations to quantify the evidence in favor of the alternative hypothesis, given the data (Wetzels & Wagenmakers, 2012).

### Software and Equipment

The task was programmed in MATLAB R2014a using the Psychophysics Toolbox extensions (Version 3; Kleiner, Brainard, & Pelli, 2007), and were displayed on a 17-in. LCD screen (1280 × 1024 pixels). The force sensor had a sampling rate of 60 Hz and a measurement accuracy of ±9.8 mN. Statistical analyses were implemented in R (Version 3.5; R Core Team, 2018; see Supplementary Table 5 in the online supplemental materials for an overview of additional packages used). The hierarchical Bayesian modeling was implemented in Stan (Carpenter et al., 2017) using the *rstan* interface package. The Method and Results sections of this article were generated from R code using the literate programming tool *knitr*. All code, data and materials are freely available through the Open Science Framework (https://osf.io/cfvxp/).

## Results

### Task Performance

**Figure 2 fig2:**
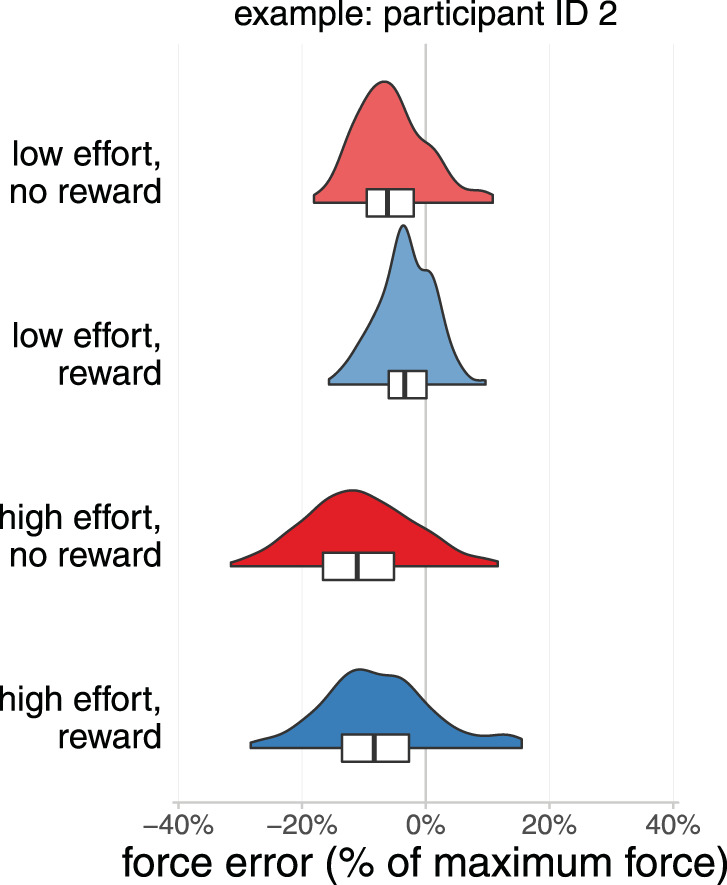
Distributions of force error by experimental conditions for a typical participant. For each distribution, the white box represents the interquartile range and the black line inside the box represents the median.

**Figure 3 fig3:**
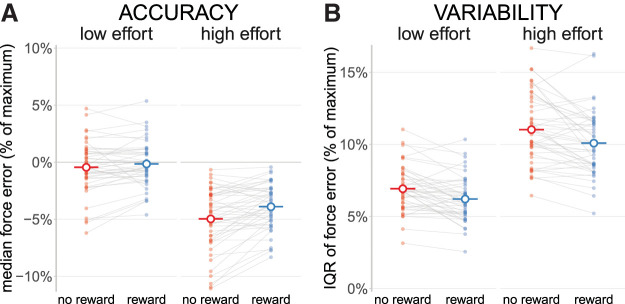
The effects of effort and reward on (A) task accuracy and (B) variability. Solid dots represent the (A) median force error or (B) interquartile range of force error for a given participant and experimental condition. The hollow dots and horizontal line segments represent the group-level mean for a given experimental condition. IQR = interquartile range.

For each subject and each condition, we obtained a distribution of force errors (see Figure 2). We examined accuracy (median force error) and variability (interquartile range) as a function of effort and reward (Figure 3; Supplementary Tables 2 and 3 in the online supplemental materials). Accuracy was lower in the high-effort condition than in the low-effort condition, as participants tended to “undershoot” the target in the high-effort condition, *F*(1, 46) = 206.01, *p* < .001, η_G_^2^ = .47. Participants were more accurate in the reward condition than in the no-reward condition, *F*(1, 46) = 15.01, *p* < .001, η_G_^2^ = .02. This reward effect was more pronounced in the high-effort condition, as indicated by a significant Effort × Reward interaction: *F*(1, 46) = 11.91, *p* < .001, η_G_^2^ = .01. However, the BF indicated that the data were equally likely under the full model including an interaction effect and the main effects model, suggesting there is no clear evidence for an interaction (BF = 0.98). [Fig-anchor fig2][Fig-anchor fig3]

Variability was greater in the high-effort condition than in the low-effort condition, *F*(1, 46) = 377.60, *p* < .001, η_G_^2^ = .52. There was reduced variability in the reward condition compared to the no-reward condition, *F*(1, 46) = 25.37, *p* < .001, η_G_^2^ = .04, but this reward effect was not different between effort conditions, as there was no significant Effort × Reward interaction: *F*(1, 46) = 0.76, *p* = .390. The BF confirmed that the data were more likely under the main effects model than the full model, providing positive evidence against an interaction effect (BF = 0.26).

Together, these results confirm that on more effortful trials, performance accuracy decreased and variability increased. In contrast, reward improved accuracy and variability in performance.

### Perception of Performance

We tested whether there was a bias in the perception of action outcomes, as found in previous studies (Wolpe et al., 2014, 2015). To this end, we measured the extent to which estimates of action outcomes were biased relative to the veridical action outcomes. We first determined whether the linear relationship between estimation error and performance error varied as a function of effort, reward, or both. We fitted a set of linear mixed models that adjusted each participant’s regression intercept and slope. The “full model” allowed for different intercepts and slopes by both effort and reward, and was the most likely model with an AIC difference to the next best model of 34.05 (Supplementary Table 4 in the online supplemental materials). We therefore report the parameters derived from the full model.

**Figure 4 fig4:**
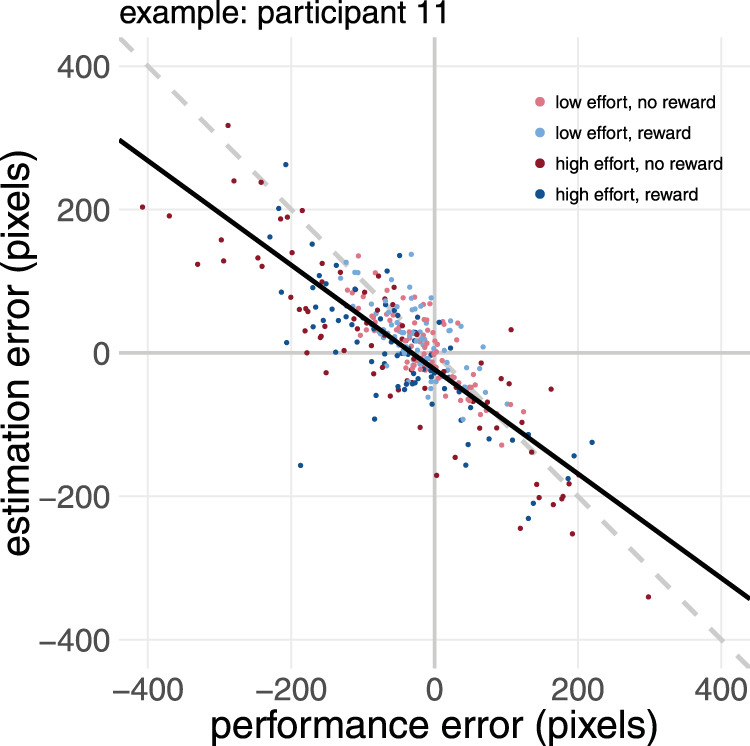
Estimation errors (difference between estimated and true ball position) plotted against performance errors (difference between true ball position and target) for a typical participant. The slope of the regression across conditions (black line) indicates the degree to which estimates of performance were biased.

Estimation errors tended to be biased, consistent with a prior centered on the target position (Figure 4; cf. Wolpe et al., 2014). The extent of this bias depended on performance error, as revealed by a strongly negative slope between estimation error and performance error (group-level β = −0.70, 95% confidence interval [−0.75, −0.66]). Individual differences in the slope ranged from −0.90 to −0.36, confirming that all participants exhibited this estimation bias.[Fig-anchor fig4]

### Prior Precision

**Figure 5 fig5:**
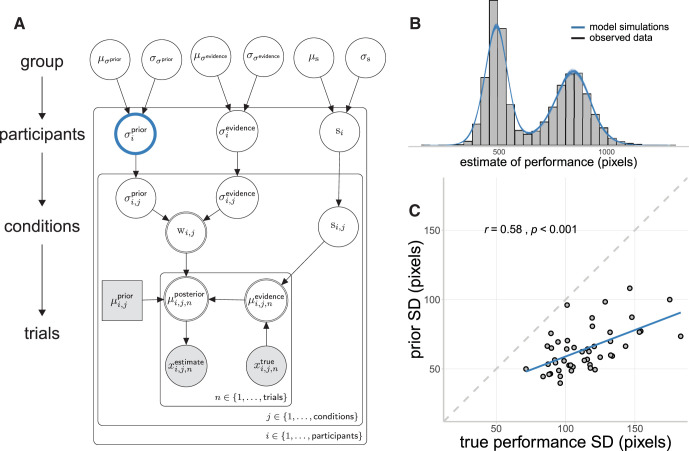
(A) The best fitting Bayesian model. Shaded nodes represent observed data whereas the white nodes represent latent variables. The rectangular node represents the target position, which is a discrete variable, whereas the remaining circular nodes represent continuous variables. The double-bordered nodes represent deterministic variables that are a function of other variables without stochastic contribution. The variable of primary interest, the standard deviation of the participant-level prior, is highlighted with a blue border. (B) Posterior predictive check for the best fitting model. The gray histogram represents the observed estimates of performance, and the overlaid blue density traces represent simulated estimates of performance drawn from the model’s posterior predictive distribution. (C) Scatterplot of the standard deviation of participant-level prior against standard deviation of performance error.

To test the hypothesis that trait apathy is associated with the precision (inverse of variance) of priors for the perception of outcomes, we used hierarchical Bayesian models (Figure 5A) to estimate the standard deviation of each participant’s prior. The model that best accounted for the data assumed that the prior was centered on the target position and included a spatial shift in sensory evidence. This model was strongly preferred over a model without a sensory evidence shift (ΔWAIC = 5,741.64, *SE*_ΔWAIC_ = 154.30) as well as a model with the prior determined by the mean and standard deviation of participants’ true performance (ΔWAIC = 7,645.63, *SE*_ΔWAIC_ = 178.99). As illustrated in Figure 5B, there was a good agreement between the observed data and simulated data drawn from the selected model’s posterior predictive distribution. We therefore proceeded with examining the posterior estimates of the participant-level prior standard deviation, *SD*.[Fig-anchor fig5]

The estimates of prior *SD* were consistently smaller than the corresponding performance error *SD*, *t*(46) = 17.18, *p* < .001 (Figure 5C). There was also a strong correlation between prior *SD* and performance error *SD*, *r*(45) = 0.58, *p* < .001. These results suggest that participants held overly precise priors that did not simply reflect the statistics of their true performance in the task.

Within participants, we allowed the prior *SD* to vary between experimental conditions (Figure 5A). We therefore examined prior *SD* as a function of effort and reward (Supplementary Figure 2 in the online supplemental materials). Given that prior *SD* was scaled to performance *SD* (Figure 5C), and performance *SD* was strongly affected by effort and reward (Figure 3B), we normalized the prior *SD* to performance *SD* (as the ratio between prior *SD* and the sum of prior *SD* and performance *SD*; Wolpe et al., 2015). This normalized prior *SD* was smaller in the high-effort condition than in the low-effort condition, *F*(1, 46) = 65.24, *p* < .001, η_G_^2^ = .19. In contrast, normalized prior *SD* was larger in the reward condition than in the no-reward condition, *F*(1, 46) = 7.00, *p* = .011, η_G_^2^ = .01. There was no significant interaction effect between effort and reward, *F*(1, 46) = 1.65, *p* = .205. The BF confirmed that the data were more likely under the main effects model than the full model, providing anecdotal evidence against an interaction effect (BF = 0.35).

**Figure 6 fig6:**
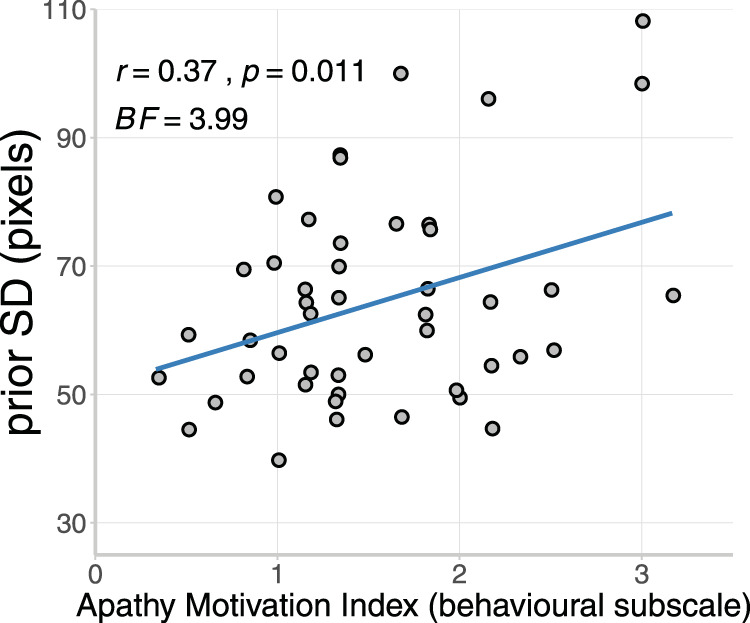
Scatterplot of the standard deviation of participant-level prior against the Apathy Motivation Index–Behavioral Activation subscale. For illustration purposes, identical questionnaire scores are jittered. BF = Bayes factor.

To test whether the precision of prior beliefs about action outcomes is associated with trait apathy, we used partial correlations of the participant-level estimates of prior *SD* and the AMI scores, adjusting for task performance variability. There was a significant partial correlation between prior *SD* and the AMI behavioral activation subscale, *r*(45) = 0.37, *p* = .011, Holm–Bonferroni corrected *p* = .045, BF = 3.99 (Figure 6). The association was positive, suggesting that individuals who were more apathetic had reduced prior precision. There were no significant partial correlations with the AMI total score, *r*(45) = 0.18, *p* = .226, BF = 0.36, emotional sensitivity subscale, *r*(45) = 0.11, *p* = .464, BF = 0.23, or social motivation subscale, *r*(45) = −0.11, *p* = .464, BF = 0.23.[Fig-anchor fig6]

Given the relationship between prior precision and sensory evidence precision in our model (see Equation 2), we also tested for partial correlations between the precision of sensory evidence and the AMI scales, adjusting for task performance variability. There were no significant correlations between *SD* of sensory evidence and the AMI total score, behavioral activation subscale, or social motivation subscale (all *p*s ≥ .411; all BFs ≤ 0.24), but there was a negative correlation with the emotional sensitivity subscale, *r*(45) = −0.38, *p* = .009, Holm–Bonferroni corrected *p* = .034, BF = 5.12.

We explored whether the effects of the experimental conditions on normalized prior *SD* depended on trait apathy, using repeated-measures analysis of covariance separately for each AMI scale (as the covariate of interest). We found no evidence for interactions between the AMI scales and effort or reward. Specifically, the effect of effort on normalized prior *SD* did not significantly interact with the AMI total score, *F*(1, 45) = 0.09, *p* = .76, behavioral activation subscale, *F*(1, 45) = 0.38, *p* = .54, emotional sensitivity subscale, *F*(1, 45) = 0.02, *p* = .89, or social motivation subscale, *F*(1, 45) = 0.02, *p* = .89. Similarly, there were no significant interactions between reward and the AMI total score, *F*(1, 45) = 0.03, *p* = .86, behavioral activation subscale, *F*(1, 45) = 1.08, *p* = .30, emotional sensitivity subscale, *F*(1, 45) = 0.02, *p* = .88, or social motivation subscale, *F*(1, 45) = 1.66, *p* = .20. The BF confirmed that the data were more likely under the main effects models (without interactions between AMI scores and the experimental conditions) than the full models (all BFs ≤ 0.05).

## Discussion

The principal result of this study is that higher trait apathy is associated with lower precision of prior beliefs about action outcomes. In the context of effortful, goal-directed actions, we confirmed that people’s perception of performance was biased, relative to the veridical action outcomes. Participants’ estimation of their action outcomes were explained by “overly precise” priors that do not simply reflect the statistics of performance. The variability of these priors was associated with trait apathy, such that more apathetic individuals tended to have less precise priors of action outcomes.

These results are consistent with a Bayesian framework of brain function, in which the brain engages in active inference on the causes of sensory inputs. Central to this model is that prior beliefs and sensory input are combined in a precision-weighted manner, so that more precise (i.e., less uncertain) information plays a stronger role in shaping action and perception. A hypothesis emerging from this framework is that a loss of prior precision leads to an impairment of goal-directed behavior (e.g., Friston et al., 2010, 2014). Our results support this hypothesis.

There is evidence that apathetic individuals tend to assign less value to a specific reward (Adam et al., 2013; Husain & Roiser, 2018; Le Bouc et al., 2016; see also Huys, Pizzagalli, Bogdan, & Dayan, 2013). When behavior is cast as active (Bayesian) inference, agents seek to maximize the evidence for their generative model of the world, rather than seeking to maximize reward as a separate construct (Friston, Daunizeau, & Kiebel, 2009). This does not make the concept of reward redundant—instead, goals and rewarding outcomes are reframed as prior beliefs that are afforded relatively high precision, so that they are preferentially fulfilled through action. Thus, our approach does not conflict with reward-based models of apathy, or the role of dopamine, but accommodates the concept of dopamine-dependent reward sensitivity within a generalized framework of behavior and perceptual inference.

Although reduced motivation for reward certainly can contribute to apathy (Adam et al., 2013), this mechanism does not fully explain the multifaceted nature of apathy in patient groups (Lansdall et al., 2017). For example, even in the absence of external prompts (such as a reward cue), apathetic patients often have difficulty in self-generating motor patterns, over and above blunted affect or cognitive dysfunction (R. Levy & Dubois, 2006). Such “auto-activation” symptoms have previously been formalized as a failure to reach a necessary activation threshold for a response (Zhang et al., 2016). Patients were surprisingly biased in favor of performing an action, but were subsequently impaired at translating this prior preference into an observed response, as indicated by a strongly reduced rate of accumulation to threshold (Zhang et al., 2016). We suggest that the diversity of symptoms associated with apathy can be understood as different expressions of a common underlying pathology: a reduction in the precision of prior beliefs about action outcomes.

Although estimates of prior precision within participants changed significantly between levels of effort and reward, the amount of change between conditions did not depend on trait apathy. This suggests that participants’ overall prior for action reflects their higher level beliefs and motivations related to trait apathy, whereas trail-to-trial changes to prior precision in light of task demands reflect lower level mechanisms of sensorimotor prediction.

In the current experiment, reward decreased the precision of priors relative to the true performance distribution. Such a strategy would promote learning in the context of expected reward, because the posterior belief will be weighted more toward the evidence than the prior. In other words, the reward manipulation facilitated learning from sensory evidence, so that trial-to-trial performance errors could be used to improve task performance. Indeed, optimizing task performance would be particularly relevant in the reward condition, as task performance in this section of the experiment determined the size of the bonus payment given after the study. In contrast to the effect of reward, we found that effort increased the precision of priors relative to the true performance distribution. This resembles the illusion of superiority (Wolpe et al., 2014) that provides a motivational advantage in the more challenging high-effort condition, when participants’ true performance tended to fall short of the target (see Figure 3).

Neuropathologies associated with apathy provide insights into the functional anatomy and candidate mechanisms of the abnormal precision of priors. Clinical apathy is associated with disruptions to frontal-subcortical circuits that are involved in self-initiated, goal-directed behavior (R. Levy & Dubois, 2006). Lesions of the prefrontal cortices have long been known to impair goal-directed behavior (Luria, 1995), and apathy is an important feature of neurodegenerative diseases affecting prefrontal regions (Chow et al., 2009; Passamonti, Lansdall, & Rowe, 2018). Lesion and neuroimaging studies have also implicated the anterior cingulate cortex and basal ganglia in apathy (Le Heron, Apps, & Husain, 2018; R. Levy & Dubois, 2006). Thus, current evidence suggests that apathy follows a disruption to fronto-striatal brain circuits.

Changes in these frontostriatal circuits have been implicated in controlling the relative precision of predictions and sensory input (Dayan & Yu, 2006; Friston et al., 2014; Moran et al., 2013). In Parkinson’s disease, the severe depletion of striatal dopamine is associated with a loss of sensory attenuation and presence of apathy, both consequences of impaired active inference (Drui et al., 2014; Macerollo et al., 2016; Santangelo et al., 2015; Wolpe et al., 2018). Indeed, individual differences in the degree of sensory attenuation were negatively related to disease severity, but positively related to dopamine medication dose (Wolpe et al., 2018). We hypothesize that neuromodulatory deficits in patients can cause a loss of prior precision relative to the sensory input, which subsequently leads to apathy. Furthermore, higher order prior beliefs about desired outcomes may fail to appropriately contextualize lower level representations about sensory input due to structural and functional abnormalities in prefrontal and temporal brain regions (Rittman et al., 2019). Further work is required to establish the synaptic and molecular basis of aberrant precision, aided by the parameterization of the precision of an individual’s prior.

Clinical studies have sought to address the extent to which apathy is distinct from other common neuropsychiatric features, particularly depression and fatigue. Conceptually, apathy does not include many of the core features of depression, such as low mood, distress and negative thoughts about oneself (Marin, 1990). Apathy can be severe in the absence of low mood or negativity, especially in neurodegenerative disorders (Kirsch-Darrow, Fernandez, Marsiske, Okun, & Bowers, 2006; Lansdall et al., 2017), and the severity of apathy and depression are not strongly correlated across patients (M. L. Levy et al., 1998). Fatigue is a complex and poorly operationalized affective state that can overlap with some of the features or descriptive terms for apathy or depression (Kuppuswamy, 2017). Few studies have directly studied the relationship between apathy and fatigue, but they can be positively correlated (Ang et al., 2017; Skorvanek et al., 2015). We suggest that Bayesian models could help to further disambiguate these behavioral phenotypes, in terms of the specific characteristics of patients’ generative models. For example, one recent model proposes that depression reflects a shift in the means of higher level prior beliefs about self-efficacy, as a consequence of prolonged interoceptive surprise (Stephan et al., 2016). Fatigue might initially represent an adaptive response to unexpected sensory input about metabolic states or bodily integrity (i.e., dyshomeostasis), in the sense that it promotes passivity and rest, while chronic dyshomeostasis leads to a generalized belief of lack of control, as in “learned helplessness” (Stephan et al., 2016). Such a model of fatigue and depression is separable from the model of apathy, as individual differences in behavior are accounted for by variation in the prior mean or prior precision, respectively. These different etiologies may co-occur within a heterogeneous group of patients, within a generalized understanding of maladaptive behavior, which nonetheless seeks to aid differential diagnosis and treatment decisions (Stephan & Mathys, 2014).

Although we propose that apathy arises from reduced precision of prior beliefs, poverty of movement could in principle also arise from excessive precision of sensory input. Such precise sensory input would “overwhelm” prior beliefs and therefore encourage passive (perceptual) inference over action. However, we found that the precision of sensory evidence was correlated only with an unexpected measure of emotional blunting (AMI emotional sensitivity) rather than the more relevant behavioral aspects of apathy. It is interesting to note the AMI behavioral activation and emotional sensitivity scales were previously found to be uncorrelated in a large normative sample, suggesting some dissociation between behavioral and emotional aspects of apathy (Ang et al., 2017). We therefore speculate that the observed correlation between sensory evidence precision and emotional blunting speaks to a separate mechanism of emotional inference (Seth & Friston, 2016), or simply results from Type I error.

There are limitations to this study. First, we examined individual differences in trait apathy in the healthy population, and the generalization to apathetic clinical disorders remains to be proven. A dimensional approach assumes that the mechanisms underlying normal variation are the same mechanisms that underlie clinical disorders (Cuthbert & Insel, 2013; Murley et al., 2019) but we recognize that apathetic patients might be qualitatively different to controls. Second, our primary results are correlational and therefore do not directly demonstrate causal mechanisms. Our results do not in themselves prove whether the precision of priors is a cause or consequence of trait apathy. Future work can adopt our approach to study apathy in the context of neurosurgical or temporary focal brain lesions (e.g., through transcranial magnetic stimulation) or pharmacological manipulations (e.g., Adam et al., 2013; Le Bouc et al., 2016; Meyniel et al., 2016). Third, we cannot comment on the variations in functional anatomy or connectivity that may determine the precision of priors in our cohort. Finally, the Bayesian framework for computational models enables relative evidences to be compared formally (Adams et al., 2016), but only between members of the subset of models tested.

In conclusion, our study suggests that apathy is associated with poor precision of prior beliefs about action outcomes. We propose that apathy can be understood as a failure to assign the necessary precision to prior beliefs about one’s action outcomes, which is required for self-initiated movement, leading to an apparent “acceptance” of the state of the world. This can be understood as satisfying an intended goal in the absence of the actual action necessary to achieve it. This approach paves the way to a common framework for understanding the causes of apathy in neurological and psychiatric disorders, and a target for novel treatment strategies.

## Context of the Research

Apathy occurs to a variable extent among healthy people, but is common in many neuropsychiatric diseases. It is typically considered as a failure of self-initiated action arising from reduced motivation, which is operationalized in terms of the cost or effort one is willing to incur for reward. We propose a new way to think about apathy, based on Bayesian inference as a fundamental property of brain function. We draw on the concept of active inference, in which action requires predictions or prior beliefs about action outcomes to be held with sufficient precision, such that priors “explain away” sensory input. The critical insight from this model is that reduced precision of priors would lead to loss of self-initiated behavior, the hallmark of apathy. We test this hypothesis using a novel visuomotor task in healthy adults, with experimental manipulation of effort and reward. Computational modeling indicates that participants use priors that are distributed narrowly around the goal, with higher precision than their true performance, and in proportion to effort and reward. Crucially, we confirm that individual differences in the prior’s precision correlate negatively with trait apathy. We suggest this approach is suitable to characterize clinical disorders, and individual differences in subclinical apathy.

## Supplementary Material

10.1037/xge0000739.supp
