# Supplementary material for: Apathy Is Associated With Reduced Precision of Prior Beliefs About Action Outcomes
Source: J Exp Psychol Gen. 2020 Feb 10;149(9):1767–77. doi: 10.1037/xge0000739 (PMC7397861; doi:10.1037/xge0000739)
Supplement: Supplementary file 1 [file xge0000739.zip › SuppFigure1.pdf]

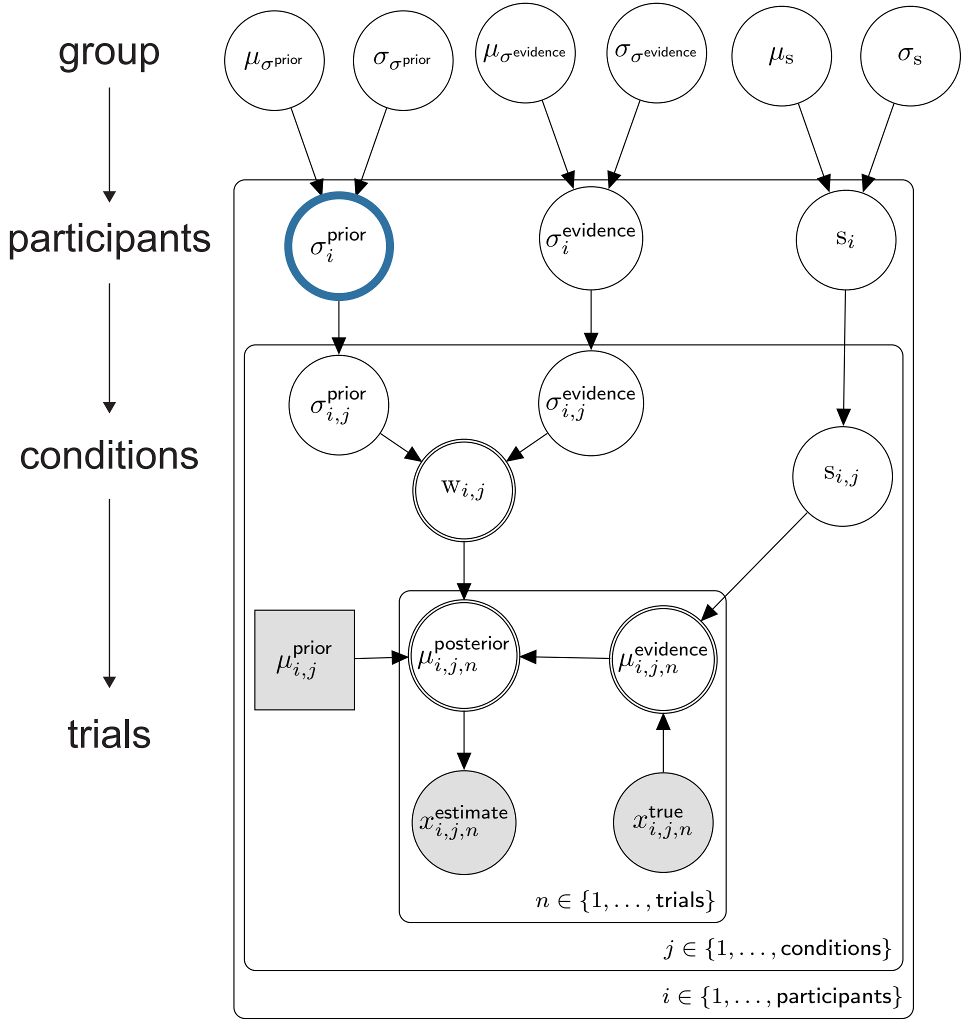

group-level prior SD:

$$\mu_{\sigma^{\text{prior}}} \sim \mathcal{N}(0, 10)$$
$$\sigma_{\sigma^{\text{prior}}} \sim \text{Cauchy}(0, 5) \qquad \sigma_{\sigma^{\text{prior}}} > 0$$

group-level sensory evidence SD:

$$\mu_{\sigma^{\text{evidence}}} \sim \mathcal{N}(0, 10)$$
$$\sigma_{\sigma^{\text{evidence}}} \sim \text{Cauchy}(0, 5) \qquad \sigma_{\sigma^{\text{evidence}}} > 0$$

group-level sensory evidence shift:

$$\mu_{\text{s}} \sim \mathcal{N}(0, 10)$$
$$\sigma_{\text{s}} \sim \text{Cauchy}(0, 5) \qquad \sigma_{\text{s}} > 0$$

---

participant-level prior SD:

$$\sigma_i^{\text{prior}} \sim \mathcal{N}(\mu_{\sigma^{\text{prior}}}, \sigma_{\sigma^{\text{prior}}})$$

participant-level sensory evidence SD:

$$\sigma_i^{\text{evidence}} \sim \mathcal{N}(\mu_{\sigma^{\text{evidence}}}, \sigma_{\sigma^{\text{evidence}}})$$

participant-level sensory evidence shift:

$$\text{s}_i \sim \mathcal{N}(\mu_{\text{s}}, \sigma_{\text{s}})$$

---

condition-level prior SD:

$$\sigma_{i,j}^{\text{prior}} \sim \mathcal{N}(\sigma_i^{\text{prior}}, 10) \qquad \sigma_{i,j}^{\text{prior}} > 0$$

condition-level sensory evidence SD:

$$\sigma_{i,j}^{\text{evidence}} \sim \mathcal{N}(\sigma_i^{\text{evidence}}, 10) \qquad \sigma_{i,j}^{\text{evidence}} > 0$$

condition-level sensory evidence shift:

$$\text{s}_{i,j} \sim \mathcal{N}(\text{s}_i, 10) \qquad -1152 < \text{s}_{i,j} < 1152$$

condition-level prior weighting:

$$\text{w}_{i,j} \leftarrow \frac{{\sigma_{i,j}^{\text{evidence}}}^2}{{\sigma_{i,j}^{\text{evidence}}}^2 + {\sigma_{i,j}^{\text{prior}}}^2}$$

---

trial-level sensory evidence mean:

$$\mu_{i,j,n}^{\text{evidence}} \leftarrow x_{i,j,n}^{\text{true}} + \text{s}_{i,j}$$

trial-level posterior mean:

$$\mu_{i,j,n}^{\text{posterior}} \leftarrow \text{w}_{i,j} \cdot \mu_{i,j}^{\text{prior}} + (1 - \text{w}_{i,j}) \cdot \mu_{i,j,n}^{\text{evidence}}$$

trial-level posterior SD:

$$\sigma_{i,j,n}^{\text{posterior}} \leftarrow \sqrt{\frac{{\sigma_{i,j}^{\text{evidence}}}^2 \cdot {\sigma_{i,j}^{\text{prior}}}^2}{{\sigma_{i,j}^{\text{evidence}}}^2 + {\sigma_{i,j}^{\text{prior}}}^2}}$$

trial-level estimate of performance:

$$x_{i,j,n}^{\text{estimate}} \sim \mathcal{N}(\mu_{i,j,n}^{\text{posterior}}, \sigma_{i,j,n}^{\text{posterior}})$$
