# Supplementary material for: Apathy Is Associated With Reduced Precision of Prior Beliefs About Action Outcomes
Source: J Exp Psychol Gen. 2020 Feb 10;149(9):1767–77. doi: 10.1037/xge0000739 (PMC7397861; doi:10.1037/xge0000739)
Supplement: Supplementary file 1 [file xge0000739.zip › Supplement_HezemansWolpeRowe_ApathyActionPriors_revision.docx]

Supplemental Material for:

Apathy is Associated with Reduced Precision of Prior Beliefs about Action Outcomes

Frank H. Hezemans, Noham Wolpe, and James B. Rowe

University of Cambridge

Supplementary Table 1

*Descriptive Statistics of Sample Demographics and Trait Apathy*

| variable | mean | SD | range |
| --- | --- | --- | --- |
| age | 24.23 | 4.84 | 18-35 |
| education | | | |
| years^a^ | 17.30 | 3.12 | 13-26 |
| degree^b^ | 1.94 | 0.89 | 1-4 |
| Apathy Motivation Index^c^ | | | |
| total | 1.34 | 0.42 | 0.5-2.39 |
| behavioural activation | 1.54 | 0.66 | 0.33-3.17 |
| social motivation | 1.48 | 0.65 | 0.33-2.83 |
| emotional sensitivity | 1.00 | 0.54 | 0.17-2.17 |

*Note*. 47 participants (24 females).
^a^ total years of formal education, including everything after kindergarten.
^b^ highest obtained degree categorised according to the British education system: 0 = GCSE (General Certificate of Secondary Education), 1 = A Levels (General Certificate of Secondary Education Advanced Level), 2 = undergraduate degree, 3 = graduate degree, 4 = postgraduate / doctorate degree.
^c^ scored on a Likert scale from 0 to 4, with higher scores indicating greater apathy (Ang, Lockwood, Apps, Muhammed, & Husain, 2017).


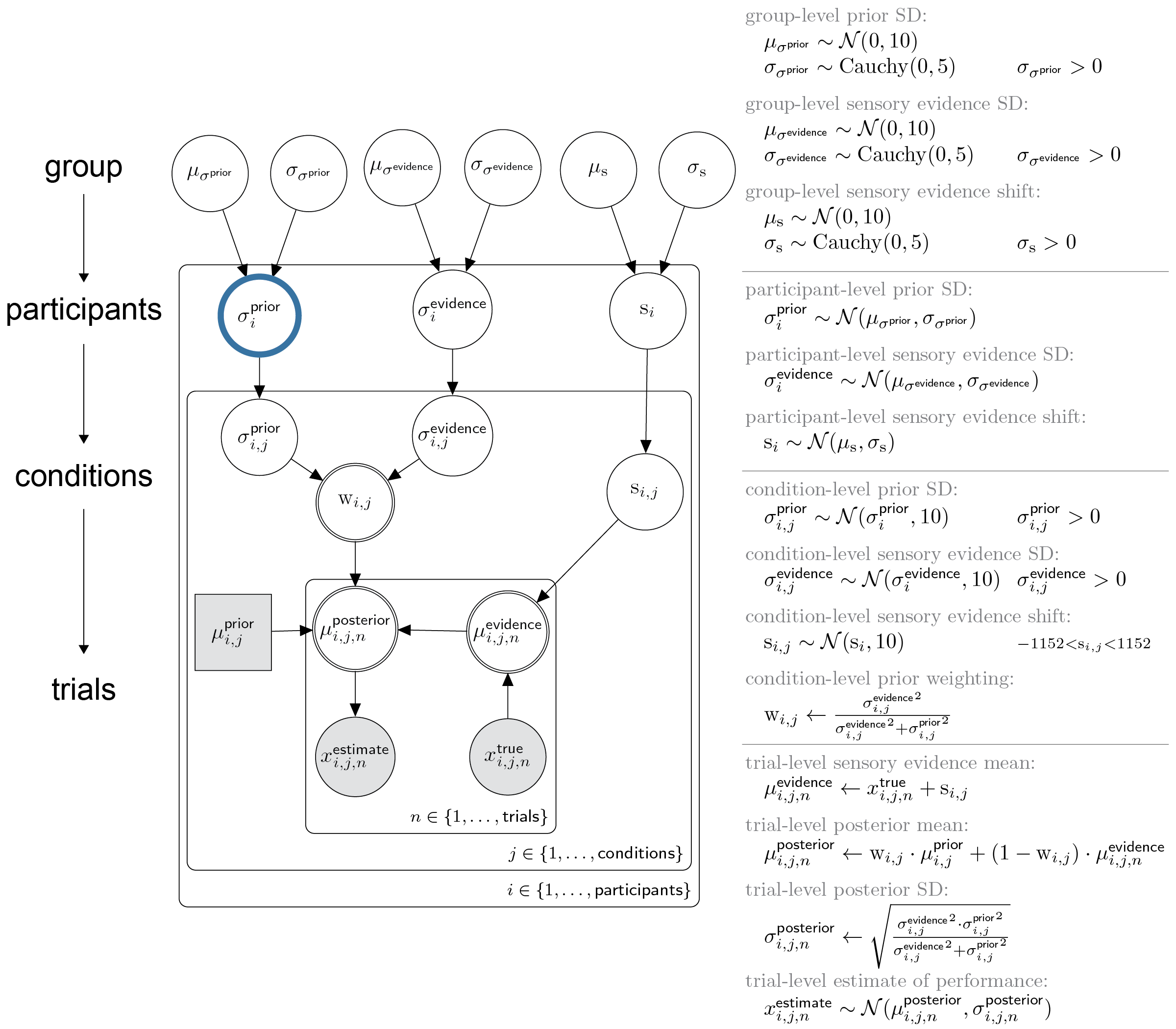


*Supplementary Figure 1.* The best fitting Bayesian model (as in Figure 5A), including the parameters’ sampling statements and functional dependencies. Participant-level parameters were sampled from latent group-level distributions, and within participants parameters were permitted to vary between conditions. Condition-level parameters were then used to compute a precision-weighted combination of the prior and trial-level sensory evidence. This trial-level posterior distribution was then used to explain the observed estimate of performance. The model is represented in plate notation: shaded nodes represent observed data whereas white nodes represent latent variables; rectangular nodes represent discrete variables whereas circular nodes represent continuous variables; and double-bordered white nodes represent deterministic variables whereas single-bordered white nodes represent stochastic variables.

Supplementary Table 2

*Descriptive Statistics of Accuracy (Median Force Error) for each Experimental Condition*

| condition |  | mean | 95% CI | *p* |
| --- | --- | --- | --- | --- |
| low effort | no reward | -0.45% | -1.09%, 0.20% |  |
|  | reward | -0.15% | -0.80%, 0.50% | .49 |
| high effort | no reward | -4.97% | -5.61%, -4.32% |  |
|  | reward | -3.90% | -4.55%, -3.25% | < .001 |

*Note*. The dependent variable, median force error, is expressed as a percentage of the participant’s maximum force. The *p*-values correspond to post-hoc Tukey’s tests comparing reward conditions within each effort condition.

Supplementary Table 3

*Descriptive Statistics of Variability (Interquartile Range of Force Error) for each Experimental Condition*

| condition |  | mean | 95% CI | *p* |
| --- | --- | --- | --- | --- |
| low effort | no reward | 6.93% | 6.36%, 7.49% |  |
|  | reward | 6.21% | 5.65%, 6.78% | .005 |
| high effort | no reward | 11.0.2% | 10.46%, 11.59% |  |
|  | reward | 10.09% | 9.52%, 10.65% | < .001 |

*Note*. The dependent variable, interquartile range of force error, is expressed as a percentage of the participant’s maximum force. The *p*-values correspond to post-hoc Tukey’s tests comparing reward conditions within each effort condition.

Supplementary Table 4

*Log Model Evidence for Linear Mixed Models of Estimation Error by Performance Error*

| model | cAIC | ΔcAIC |
| --- | --- | --- |
| basic | 30687.59 | 1913.25 |
| varying intercepts | | |
| effort | 28985.04 | 210.71 |
| reward | 30569.43 | 1795.10 |
| effort & reward | 28808.39 | 34.05 |
| varying slopes | | |
| effort | 30529.66 | 1755.33 |
| reward | 30620.91 | 1846.57 |
| effort & reward | 30481.70 | 1707.37 |
| varying intercepts & slopes | | |
| effort | 28973.41 | 199.08 |
| reward | 30530.18 | 1755.84 |
| **effort & reward** | **28774.34** | **0.00** |

*Note*. cAIC = conditional Akaike Information Criterion (Greven & Kneib, 2010), ΔcAIC = [cAIC – min(cAIC)]. The ‘basic’ model allowed for varying intercepts and slopes between participants, but did not take into account the experimental conditions of effort and reward. The nine remaining models allowed for further adjustments to the intercept, slope, or both by effort, reward, or both. The favoured model, with varying intercepts and slopes by effort and reward, is highlighted in bold.

*
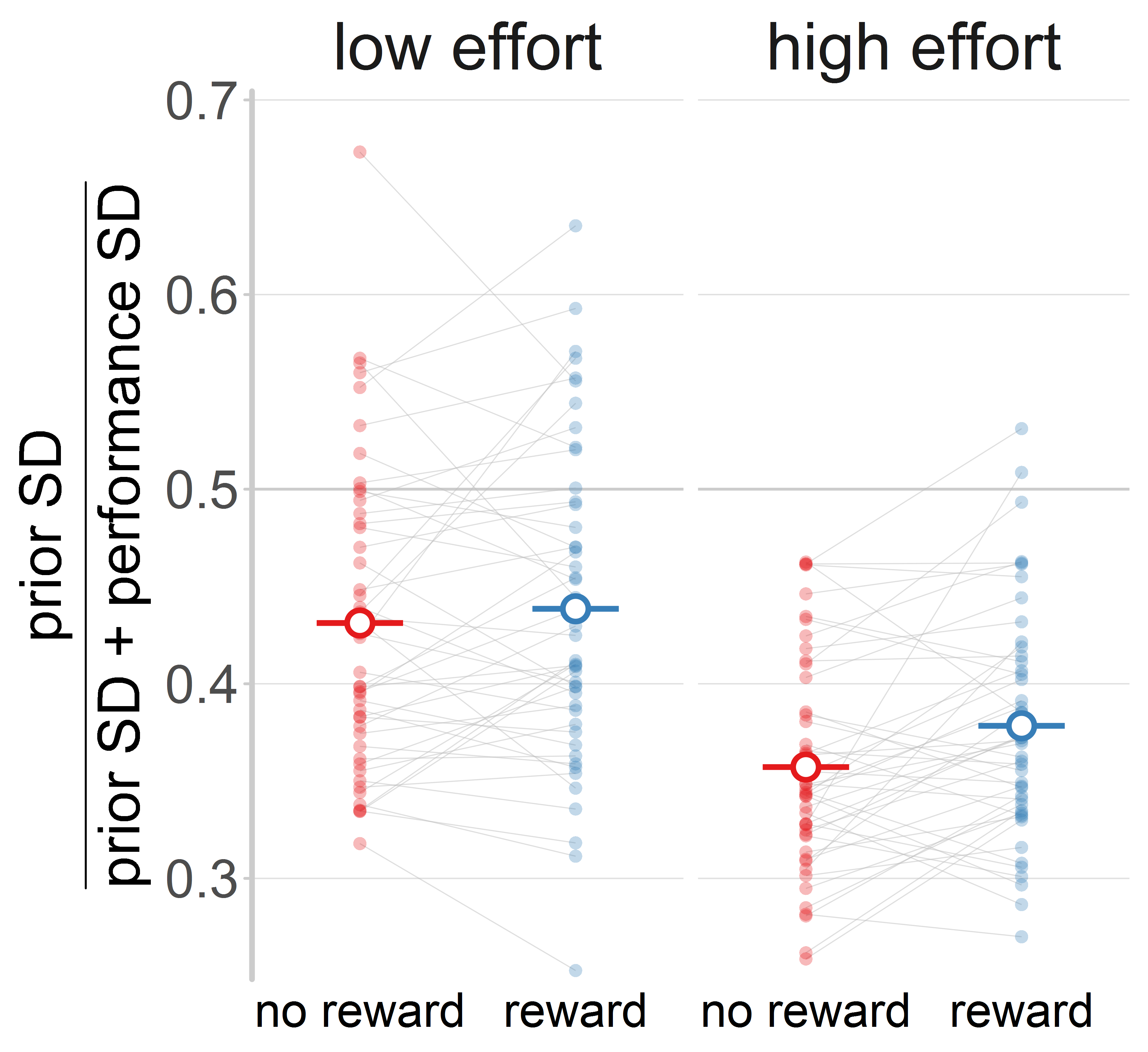
*

*Supplementary Figure 2*. The effects of effort and reward on the standard deviation of the prior, normalised to the standard deviation of performance error (Wolpe, Nombela, & Rowe, 2015). Values smaller than 0.5 represent priors that are more precise than the corresponding performance distribution. Solid dots represent individual participants, whereas the hollow dots and horizontal line segments represent the group-level mean for a given experimental condition.

Supplementary Table 5

*R Packages used for Statistical Analysis*

| package | usage | citation |
| --- | --- | --- |
| tidyverse (version 1.2.1) | data organisation and visualisation | (Wickham, 2017) |
| pwr (version 1.2-2) | power calculations for correlation test | (Champely, 2018) |
| afex (version 0.21-2) | ANOVA | (Singmann, Bolker, Westfall, & Aust, 2017) |
| BayesFactor (version 0.9.12-4.2) | Bayes factor for ANOVA and regression models | (Morey & Rouder, 2018) |
| emmeans (version 1.2.2) | estimated marginal means and post-hoc Tukey’s tests | (Lenth, 2018) |
| lme4 (version 1.1-20) | linear mixed models | (Bates, Mächler, Bolker, & Walker, 2015) |
| cAIC4 (version 0.5) | conditional Akaike information criterion for linear mixed models | (Säfken, Rügamer, Kneib, & Greven, 2018) |
| merTools (version 0.4.1) | extract and organise parameter estimates from linear mixed model | (Knowles & Frederick, 2018) |
| rstan (version 2.18.2) | hierarchical Bayesian modelling | (Stan Development Team, 2018) |
| loo (version 2.0.0) | widely applicable information criterion for Bayesian models | (Vehtari, Gabry, Yao, & Gelman, 2018) |
| tidybayes (version 1.0.4) | extract and organise parameter estimates from Bayesian models | (Kay, 2019) |
| knitr (version 1.20) | generate methods and results sections from R code | (Xie, 2018) |

Supplemental References

Ang, Y.-S., Lockwood, P., Apps, M. A. J., Muhammed, K., & Husain, M. (2017). Distinct Subtypes of Apathy Revealed by the Apathy Motivation Index. *PLOS ONE*, *12*(1), e0169938. https://doi.org/10.1371/journal.pone.0169938

Bates, D., Mächler, M., Bolker, B., & Walker, S. (2015). Fitting Linear Mixed-Effects Models Using lme4. *Journal of Statistical Software*, *67*(1). https://doi.org/10.18637/jss.v067.i01

Champely, S. (2018). *pwr: Basic Functions for Power Analysis*. Retrieved from https://CRAN.R-project.org/package=pwr

Greven, S., & Kneib, T. (2010). On the behaviour of marginal and conditional AIC in linear mixed models. *Biometrika*, *97*(4), 773–789. https://doi.org/10.1093/biomet/asq042

Kay, M. (2019). *tidybayes: Tidy Data and Geoms for Bayesian Models*. Retrieved from http://mjskay.github.io/tidybayes/

Knowles, J. E., & Frederick, C. (2018). *merTools: Tools for Analyzing Mixed Effect Regression Models*. Retrieved from https://CRAN.R-project.org/package=merTools

Lenth, R. (2018). *emmeans: Estimated Marginal Means, aka Least-Squares Means*. Retrieved from https://CRAN.R-project.org/package=emmeans

Morey, R. D., & Rouder, J. N. (2018). *BayesFactor: Computation of Bayes Factors for Common Designs*. Retrieved from https://CRAN.R-project.org/package=BayesFactor

Säfken, B., Rügamer, D., Kneib, T., & Greven, S. (2018). *Conditional Model Selection in Mixed-Effects Models with cAIC4*. Retrieved from http://arxiv.org/abs/1803.05664

Singmann, H., Bolker, B., Westfall, J., & Aust, F. (2017). *afex: Analysis of Factorial Experiments*. Retrieved from https://CRAN.R-project.org/package=afex

Stan Development Team. (2018). *RStan: the R interface to Stan*. Retrieved from http://mc-stan.org/

Vehtari, A., Gabry, J., Yao, Y., & Gelman, A. (2018). *loo: Efficient leave-one-out cross-validation and WAIC for Bayesian models*. Retrieved from https://CRAN.R-project.org/package=loo

Wickham, H. (2017). *tidyverse: Easily Install and Load the “Tidyverse.”* Retrieved from https://CRAN.R-project.org/package=tidyverse

Wolpe, N., Nombela, C., & Rowe, J. B. (2015). Dopaminergic modulation of positive expectations for goal-directed action: evidence from Parkinson’s disease. *Frontiers in Psychology*, *6*. https://doi.org/10.3389/fpsyg.2015.01514

Xie, Y. (2018). *knitr: A General-Purpose Package for Dynamic Report Generation in R*. Retrieved from https://yihui.name/knitr/
