# Supplementary figures and images for: Apathy Is Associated With Reduced Precision of Prior Beliefs About Action Outcomes
Source: J Exp Psychol Gen. 2020 Feb 10;149(9):1767–77. doi: 10.1037/xge0000739 (PMC7397861; doi:10.1037/xge0000739)

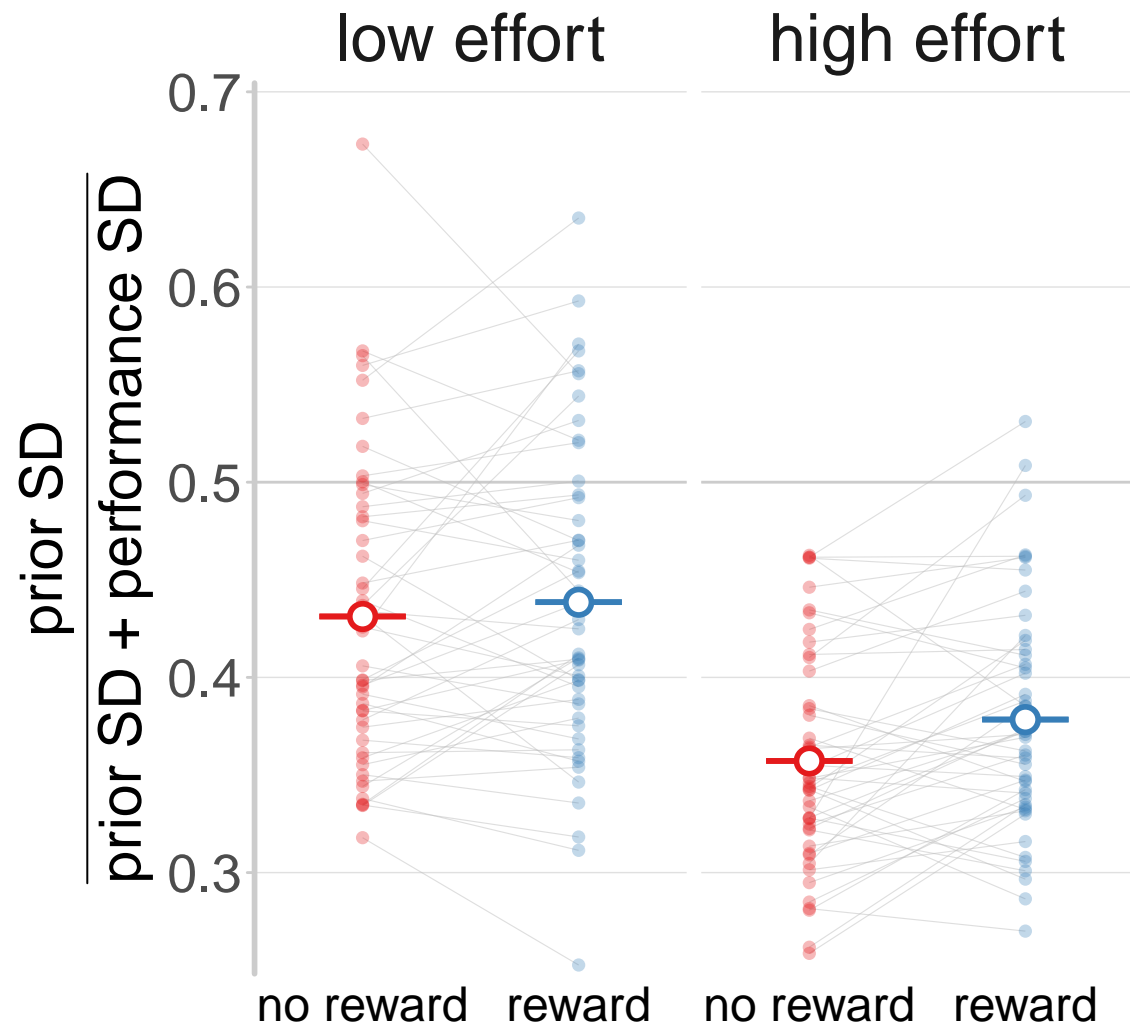

Supplement: Supplementary file 1 [file xge0000739.zip › SuppFigure2.pdf]
